# The Relationship Between Personality Traits, Psychopathological Symptoms, and Problematic Internet Use: A Complex Mediation Model

**DOI:** 10.2196/11837

**Published:** 2019-04-26

**Authors:** Beatrix Koronczai, Gyöngyi Kökönyei, Mark D Griffiths, Zsolt Demetrovics

**Affiliations:** 1 Department of Developmental and Clinical Child Psychology Institute of Psychology Eötvös Loránd University Budapest Hungary; 2 Department of Clinical Psychology and Addiction Institute of Psychology Eötvös Loránd University Budapest Hungary; 3 SE-NAP2 Genetic Brain Imaging Migraine Research Group Semmelweis University Budapest Hungary; 4 Department of Pharmacodynamics Faculty of Pharmacy Semmelweis University Budapest Hungary; 5 Psychology Division Nottingham Trent University Nottingham United Kingdom

**Keywords:** problematic internet use, personality, psychopathology, psychopathological symptoms

## Abstract

**Background:**

There are many empirical studies that demonstrate the associations between problematic internet use, psychopathological symptoms, and personality traits. However, complex models are scarce.

**Objective:**

The aim of this study was to build and test a mediation model based on problematic internet use, psychopathological symptoms, and personality traits.

**Methods:**

Data were collected from a medical addiction center (43 internet addicts) and internet cafés (222 customers) in Beijing (mean age 22.45, SD 4.96 years; 239/265, 90.2% males). Path analysis was applied to test the mediation models using structural equation modeling.

**Results:**

Based on the preliminary analyses (correlations and linear regression), two different models were built. In the first model, low conscientiousness and depression had a direct significant influence on problematic internet use. The indirect effect of conscientiousness—via depression—was nonsignificant. Emotional stability only affected problematic internet use indirectly, via depressive symptoms. In the second model, low conscientiousness also had a direct influence on problematic internet use, whereas the indirect path via the Global Severity Index was again nonsignificant. Emotional stability impacted problematic internet use indirectly via the Global Severity Index, whereas it had no direct effect on it, as in the first model.

**Conclusions:**

Personality traits (ie, conscientiousness as a protective factor and neuroticism as a risk factor) play a significant role in problematic internet use, both directly and indirectly (via distress level).

## Introduction

Most empirical studies to date have found a positive association between problematic internet use and psychopathological symptoms in normal samples of both adolescents [1-5] and adults [6-11]. A few studies have examined this relationship among clinical samples (ie, among diagnosed internet addicts), comparing them to healthy control groups [2,12,13] or clinical control groups [14,15]. The results of sampling from both clinical and normal populations have demonstrated an increased level of psychopathological symptoms among problematic internet users. When predictor variables have been examined for problematic internet use, findings have also been consistent. In most studies, depressive [1,14,10,12,13,5,15] and obsessive-compulsive symptoms [8,1,9,14,13,4,15] have been found to be the most significant predictors of problematic internet use.

Additionally, several studies have reported important predictors of problematic internet use (or they are present at a more extensive level in the group of problematic internet users), including hostility [1,9,13,4,5], anxiety [10,12,5,13], and interpersonal sensitivity [8,1,15]. One longitudinal study [16] has provided indicative data concerning the cause-and-effect between problematic internet use and psychopathological symptoms. The results suggested that obsessive-compulsive symptoms are predictors of internet addiction, whereas increased levels of depression, anxiety, hostility, interpersonal sensitivity, and psychoticism are consequences of internet addiction.

A meta-analysis by Kayis et al [17] that evaluated 12 studies found that all five main factors of the Big Five model correlated with problematic internet use. More specifically, agreeableness, openness to experience, extraversion, and conscientiousness were negatively associated with internet addiction, whereas neuroticism was positively associated with internet addiction. In general, the relationship between neuroticism and problematic internet use appears the most established. Neuroticism has been positively associated with (1) problematic internet use in all empirical research to date in correlational studies (eg, [18-20]), (2) comparison of groups of internet addicts and controls (eg, [21,22]), and (3) regression analyses (eg, [23,19]). This association is also found in research assessing neuroticism by questionnaires based on (1) Eysenck’s three-factor theory (eg, [24-33]) and (2) Zuckerman’s five-factor model (eg, [34]). Similarly, studies have also reported an association between low agreeableness and internet addiction (eg, [21,23,18,20]) and low conscientiousness and internet addiction (eg, [18,22,20]).

The direction of the association between extraversion and problematic internet use is controversial. Some studies have demonstrated a positive relationship with more symptoms of internet addiction associated with higher extraversion (eg, [21,18,20]). However, another study reported a negative association with a higher level of problematic internet use correlated with higher introversion [22]. Regarding Eysenck’s three-factor model, introversion has also been related to problematic internet use in some cases (eg, [35,25,13,31]). Additionally, Zuckerman’s sociability and activity factors (which may correspond with extraversion), have also been found to correlate negatively with internet addiction [34]. Similar incoherence has been found in the case of openness to experience. One study reported an association between problematic internet use and low openness to experience [21], whereas another reported a positive association between internet addiction and openness to experience [21].

To date, there have been relatively few mediation or moderation models examining the complex associations and interactions between personality traits, internet addiction, and other variables. Researchers have examined the associations between specific personality traits and problematic internet use via coping strategies [21]. Additionally, personality traits have been shown to mediate the impact of time spent online on internet addiction [36]. Kuss et al [23] also demonstrated that the interactions between different online activities and personality traits affect the likelihood of becoming an internet addict.

To the authors’ knowledge, only two studies have tested complex models including variables comprising personality, psychopathology, and problematic internet use. One of them [37] presented a model in which personality was characterized in terms of the behavioral inhibition and behavioral activation systems, and depression, impulsivity, and anxiety were considered psychopathologies. The study found that both personality variables influenced internet addiction and that the effect was mediated by anxiety and/or depression and/or impulsivity in different ways. Floros et al [38] described a path model analysis in which personality traits were conceptualized by Zuckerman’s alternative five-factor model, and psychopathological symptoms were assessed using the global indexes of the 90-item Symptom Checklist. In this model, personality traits and defense style both had an effect on internet addiction, and internet addiction predicted psychopathological symptoms (versus the reverse).

**Figure 1:**
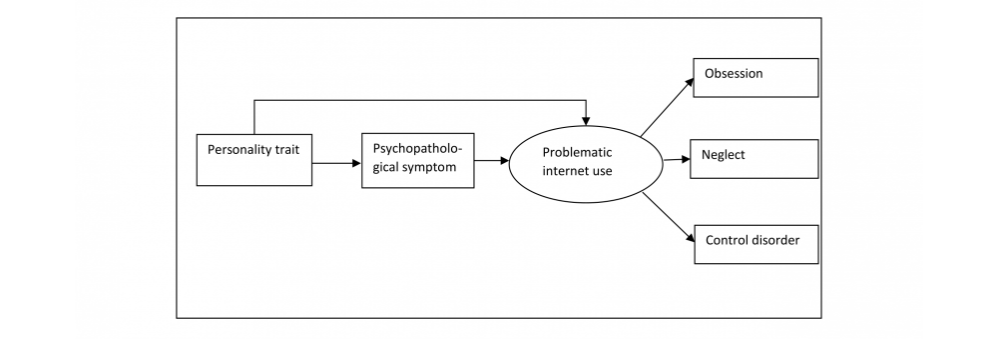
Proposed mediation model.

In summary, there are many empirical studies that demonstrate associations between internet addiction and psychopathological symptoms, and between internet addiction and personality traits. However, further analysis is needed on the complex effects and models. Given the lack of research, the aim of this study was to build and test a mediation model that examined personality factors, psychopathological symptoms, and problematic internet use within a single complex model (see Figure 1). The investigation of complex effects is relevant in particular for problematic internet users because the outcomes might facilitate the focus of their treatment.

## Methods

### Participants and Procedure

The data for this sample were collected from two samples of intensive internet users. Although the two samples appear to be distinct, this sampling method can be explained by specific Chinese circumstances. In an internet addiction clinic, the patients are not a simple treatment-seeking population because the young internet users often are delegated (and sometimes forced) to enter treatment by their parents. Based on some prior reports (eg, [39,40]), intensive users with a high risk for problematic internet use can be found in internet cafés. Sample 1 (the clinical group) consisted of diagnosed internet addicts who were hospitalized at an addiction medical center in Beijing that specializes in the treatment of problematic internet users. Each patient admitted to the hospital and diagnosed for problematic internet use was included in the sample during the 9 months of the study. In the case of patients younger than 18 years, both the patients and their parents were informed about the study goals and were asked to provide informed consent. Participation was voluntary, and the questionnaires were completed anonymously. Sample 2 (the internet café group) consisted of customers of internet cafés in the Chaoyang District of Beijing. Managers of 15 internet cafés were asked for permission to carry out the data collection, and 13 agreed. Each of the 13 cafés were visited three times. During data collection, each customer was invited to participate in the study and approximately 10% (222/1850) agreed to participate. A small gift was offered as recompense for participation in the study (ie, money for 2-hour internet use or a soft drink; appoximately US $1.50) was offered. The customers completed the questionnaires on site but via an online survey. Participation in the research was voluntary and anonymous. The participants could read information about the study and provide informed consent prior to completing the questionnaire. The study protocol was approved by the Institutional Review Board of Eötvös Loránd University, Budapest. The final sample consisted of 43 diagnosed internet addicts (42 males, 1 female) and 222 internet café customers (197 males, 25 females).

### Measures

#### Demographic Data and Internet Use Characteristics

Basic personal demographic information and other questions were asked about the location, the duration, the frequency, and the purpose of the participants’ internet use.

#### Problematic Internet Use Questionnaire-9

The Chinese version of Problematic Internet Use Questionnaire (PIUQ-9) [41] consists of three factors (obsession, neglect, and control disorder) with three items relating to each factor. The obsession subscale relates to mental withdrawal symptoms caused by the lack of internet use (eg, “How often do you feel tense, irritated, or stressed if you cannot use the internet for as long as you want to?”). The neglect subscale contains items related to difficulties in controlling internet use (eg, “How often do you spend time online when you’d rather sleep?”). The control disorder subscale relates to difficulties in controlling internet use (eg, “How often do you try to conceal the amount of time spent online?”). Participants use a 5-point Likert scale to estimate the extent to which each given statement is true to them. The scale ranges from 9 to 45; the maximum scores are 15 for the subscales. Higher scores indicate more symptoms of problematic internet use.

#### Big Five Mini-Markers

The Big Five Mini-Markers scale (BFI) [42] is a shortened version of Goldberg’s unipolar Big-Five Markers [43] and consists of 40 adjectives. Participants evaluate every adjective according to how well it describes them on a 9-point Likert scale. It has five factors that assess the participants’ overall personality (ie, extraversion, agreeableness, conscientiousness, emotional stability, and intellect/openness). In all subscales, higher scores indicate a higher level of that specific personality characteristic. The maximum score on each subscale is 72.

#### Brief Symptom Inventory

The Brief Symptom Inventory (BSI) test [44] is a shortened version of the Symptom Checklist-90-R [45]. It consists of 53 items; participants assess how much the symptoms bothered them the previous week on a 5-point Likert scale. The scale lists the clinically relevant psychological symptoms that are indicators of emotional distress. The items include nine dimensions: somatization, obsessive-compulsive symptoms, interpersonal sensitivity, depression, anxiety, hostility, phobic anxiety, paranoia, and psychoticism. For all the subscales, higher scores indicate more psychopathological symptoms. In addition, a global index was used, namely the Global Severity Index, which is the mean of all the items. The maximum score on the interpersonal sensitivity subscale is 20; 25 on the hostility, phobic anxiety, paranoid ideation, and psychoticism subscales; 30 on the obsessive-compulsive symptoms, depression, and anxiety subscales; 35 on the somatization subscale, and 5 on the Global Severity Index.

### Statistical Analysis

For statistical analyses, SPSS version 23.0 and Mplus version 7.11 statistical software packages were used. In addition to the mean and standard deviation of the scales, Cronbach alphas were calculated as indexes of internal consistency, which were considered good if the values were at least .70 [46]. Correlational analysis and regression analysis were also applied. Based on these results, path analysis was used to test the mediation models with structural equation modeling using maximum likelihood estimation robust to nonnormality [47]. To evaluate the overall fit of the models, the absolute fit index (chi-square test), the comparative fit index (CFI), the Tucker-Lewis index (TLI) or nonnormed fit index, and the root mean square error approximation (RMSEA) were used. The CFI and TLI are related to the total variance accounted by the model, with values higher than 0.95 indicating a good fit, and values below 0.90 indicating a poor fit [48]. The RMSEA is related to the variance of the residuals, and values below 0.08 are considered an acceptable fit, while values below .05 indicate a good fit. Closeness of model fit (CFit) using RMSEA (CFit of RMSEA) evaluating the statistical deviation of RMSEA from the value 0.05 is also reported. Nonsignificant probability values (*P*>.05) indicate acceptable fit. However, some methodologists suggest values larger than *P*>.50 [48].

## Results

### Descriptive Statistics

The mean age of participants was 22.45 (SD 4.96) years in the total sample, 17.9 (SD 0.42) years in the clinical group, and 23.47 (SD 4.77) years in the internet café group. The age difference between the two samples was statistically significant (*t*_217_=10.056, *P*<.001). The time spent on the internet for the purpose of studying or working is presented in Table 1. Approximately one-third of the sample used the internet for studying or working 3 to 4 hours a day. This represented the largest category out of the six options given among all internet use. Approximately 10% of the participants declared that they spent more than 8 hours a day online for the purpose of studying or working. Table 1 also shows the time spent on the internet for purposes other than studying or working and the pattern was similar. Two-thirds of the participants used the internet for entertainment 1 to 2 hours or 3 to 4 hours a day, and slightly less than 10% used the internet for entertainment for more than 8 hours a day.

The clinical group reported higher total PIUQ score and higher scores on the neglect factor than the internet café group. Also, a significant difference was found between the clinical group and the internet café group according to BFI intellect/openness (see Table 2). The effect size for differences in the total PIUQ score and for the neglect factor was small (Cohen *d*=0.41), but medium and large for the PIUQ neglect factor (Cohen *d*=0.64) and intellect/openness (Cohen *d*=0.87).

Correlations between the variables of the study are reported in Multimedia Appendix 1.

Based on previous results [49], 22 points (out of 45) was defined as a cut-off point of the PIUQ-9, which created two categories of internet users (problematic and nonproblematic users) The proportion of problematic internet users was 37% (16/43) in the clinical group and 31.9% (71/222) in the internet café group. Applying linear regression, symptoms which remained in significant relationships were tested with problematic internet use (which was a continuous variable) after controlling for the effects on one another. In addition to the sample category that the participants were in, the increased levels of obsessive-compulsive and depressive symptoms contributed significantly to an explanation of the variance of total scores (see Table 3).

**Figure 2:**
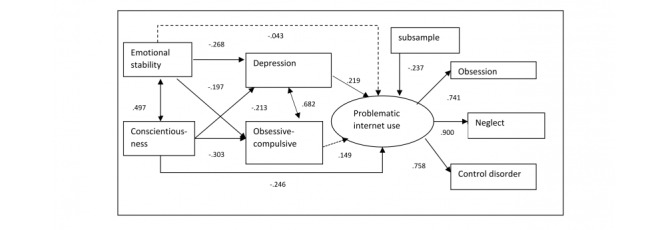
The mediation model and standardized path coefficients. Dashed arrows indicate nonsignificant path coefficients; continuous arrows indicate significant paths.

Based on the preliminary analyses (correlations and linear regression), a model was built to investigate the relationships between problematic internet use, personality traits, and psychopathological symptoms (see Figure 2). It was assumed that depressive and obsessive-compulsive symptoms mediated the relationship between personality traits (emotional stability, conscientiousness) and problematic internet use (defined here as a latent variable). The subsample variable was also added to the model because there was a difference between the two subsamples in the PIUQ total score. Additionally, after performing linear regression, the subsample variable was significant in predicting the PIUQ score.

**Table 1:** Time spent on the internet for working/studying and other purposes (N=265).

Hours per day	Working or studying, n (%) (n=262)	Other purposes, n (%) (n=261)
<1	59 (22.5)	37 (14.2)
1-2	52 (19.8)	82 (31.4)
3-4	73 (27.9)	81 (31.0)
5-6	26 (9.9)	27 (10.3)
7-8	21 (8.0)	11 (4.2)
>8	31 (11.8)	23 (8.8)

**Table 2:** Means (standard deviations) and differences by group with Cronbach alphas.

Scale^a^		Cronbach alpha	Total sample (N=265), mean (SD)	Clinical group (n=43), mean (SD)	Internet café group (n=222), mean (SD)	*t* value (df)	*P* value
**PIUQ-9**							
	Total	.848	20.10 (8.16)	23.15 (9.75)	19.53 (7.73)	2.223 (252)	.03
	Obsession	.749	5.74 (3.09)	6.28 (3.55)	5.63 (2.98)	1.262 (260)	.21
	Neglect	.713	7.20 (3.14)	8.95 (3.47)	6.87 (3.01)	3.966 (256)	<.001
	Control	.886	7.12 (3.03)	7.79 (3.67)	7.00 (2.88)	1.554 (258)	.12
**BSI**							
	Somatization	.840	9.80 (3.95)	9.71 (3.59)	9.81 (4.03)	0.157 (243)	.88
	Obsessive-compulsive	.817	10.77 (4.54)	10.29 (4.07)	10.86 (4.64)	0.730 (241)	.47
	Interpersonal sensitivity	.791	7.38 (3.59)	6.93 (3.09)	7.47 (3.68)	0.884 (243)	.38
	Depression	.871	10.11 (5.00)	9.66 (3.86)	10.21 (5.20)	0.777 (243)	.44
	Anxiety	.826	8.51 (3.75)	8.88 (3.84)	8.44 (3.74)	0.688 (243)	.49
	Hostility	.790	7.85 (3.39)	8.22 (3.23)	7.78 (3.42)	0.759 (243)	.45
	Phobic anxiety	.712	7.13 3.04)	6.88 (2.53)	7.18 (3.14)	0.582 (244)	.56
	Paranoid ideation	.772	7.68 (3.30)	7.74 (3.26)	7.67 (3.32)	0.140 (245)	.89
	Psychoticism	.775	7.73 (3.46)	8.07 (3.68)	7.66 (3.42)	0.694 (243)	.49
Global Severity Index		.970	1.57 (0.60)	1.56 (0.54)	1.58 (0.61)	0.145 (239)	.89
**BFI**							
	Extraversion	.540	43.95 (7.87)	44.98 (10.83)	43.74 (7.14)	0.707 (247)	.48
	Agreeableness	.711	51.24 (8.79)	53.02 (9.73)	50.88 (8.57)	1.441 (247)	.15
	Conscientiousness	.712	44.20 (8.68)	44.69 (10.35)	44.10 (8.33)	0.349 (247)	.73
	Emotional Stability	.734	45.61 (9.78)	47.05 (12.60)	45.32 (9.12)	0.845 (249)	.40
	Intellect/Openness	.734	46.03 (8.87)	52.43 (9.33)	44.73 (8.20)	5.412 (247)	<.001

^a^BFI: Big Five Inventory; BSI: Brief Symptom Inventory; PIUQ-9: Problematic Internet Use Questionnaire.

**Table 3:** Linear regression for prediction of problematic internet use (*R*^2^=.239).

Independent variable		Standardized β	*P* value
Sex (0: male, 1: female)		–.013	.83
Subsample (0: clinical, 1: internet café)		–.198	.001
**BSI^a^**			
	Somatization	–.023	.82
	Obsessive-compulsive	.258	.01
	Interpersonal sensitivity	–.020	.85
	Depression	.362	.003
	Anxiety	.005	.97
	Hostility	.096	.34
	Phobic anxiety	–.029	.79
	Paranoid ideation	–.130	.22
	Psychoticism	–.087	.47

^a^BSI: Brief Symptom Inventory.

**Figure 3:**
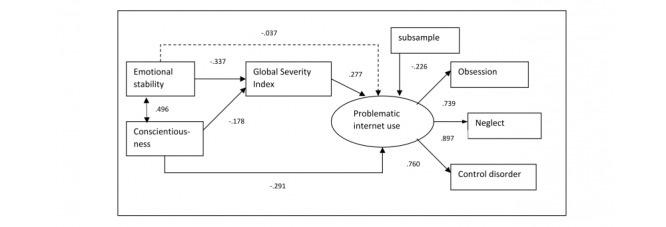
Mediation model with Global Severity Index. Dashed arrows indicate nonsignificant path coefficients; continuous arrows indicate significant paths.

The goodness-of-fit indexes of the mediation model were appropriate (χ^2^_14_^=^14.5, *P*=.28; CFI=0.995, TLI=0.991, RMSEA=0.026, 90% CI 0.000-0.068, CFit=0.792). Low conscientiousness and depression had a direct significant effect on problematic internet use, whereas the direct effects of emotional stability and obsessive-compulsive symptoms were nonsignificant. Both emotional stability and low conscientiousness significantly explained the symptoms of depression and obsessive-compulsive disorder. This meant that low conscientiousness directly impacted problematic internet use. However, the indirect effect of low conscientiousness—via depression—was nonsignificant (standardized indirect effect=–0.047, *P=*.11). Emotional stability only affected problematic internet use indirectly, via depressive symptoms (standardized indirect effect=–0.059, *P=*.03). The impact of the sample category on problematic internet use was significant (see Figure 2). The participants in the clinical sample had higher scores on the PIUQ-9 compared to the internet café sample. The model explained 32.5% of the total variance of problematic internet use. Given that all the psychopathological symptoms positively correlated with problematic internet use, another mediation model was tested in which the Global Severity Index was used instead of the individual symptoms (see Figure 3).

The goodness-of-fit indexes of the second mediation model were good (χ^2^_11_=16.2, *P*=.13; CFI=0.985, TLI=0.975, RMSEA=0.042, 90% CI: 0.000-0.083). Low conscientiousness had a direct effect on problematic internet use, whereas the indirect path via the Global Severity Index was nonsignificant (standardized indirect effect=–0.049, *P=*.10). Emotional stability impacted problematic internet use indirectly via the Global Severity Index (standardized indirect effect=–0.094, *P*<.001), whereas it had no direct effect on it. The model explained 28.9% of the total variance of problematic internet use.

## Discussion

The results of this study demonstrated that both samples showed much higher levels of problematic internet use than those observed in normal populations (7.1% in Asia) [50]. Although this was expected in the clinical sample, the similar prevalence among those recruited from internet cafés was nonevident at first sight. However, internet cafés have a special position in Chinese internet culture [51-54]. In internet cafés, young people (mostly males, younger than 30 years) play online games, chat online, and watch movies. It is perhaps not surprising that the prevalence of internet addiction is high among the patronage of internet cafés [39,40,55]. Furthermore, Griffiths et al [56] noted that parents in Southeast Asian countries appear to pathologize any behavior of their children that takes time away from educational pursuits and family. This tendency—the parents tend to feel anxious due to their (mainly male) children’s school performance—might lead to more vigilance for any symptoms of problematic internet use and to seeking help for their adolescents. Psychiatrists interviewed the problematic internet users in this study; however, the diagnosis of internet addiction is not official, and the scale used to assess problematic internet use is not based on official diagnostic criteria. Consequently, there might be a discrepancy in the level of symptoms based on currently used clinical interviews and the scale used in this study (PIUQ).

Based on the outcomes of the preliminary statistical analyses, low conscientiousness and emotional stability negatively correlated with problematic internet use. These findings are congruent with previous results reported in the literature on problematic internet use [21,23,18,19,22,20]. In their meta-analysis, Kotov et al [57] found in adults that high neuroticism (equivalent to low emotional stability) and low conscientiousness were also associated with anxiety, depression, and substance use disorders.

Neuroticism was the strongest correlate among the five personality traits, and low conscientiousness was the second trait to have a strong and consistently negative effect size. In another study [58], similar findings were reported. Extraversion, low conscientiousness, and low emotional stability had the strongest predictive values on psychopathological symptoms. In a large sample of psychosomatic outpatients [59], the level of neuroticism was a differentiating factor between the clinical and nonclinical samples with a large effect size. Additionally, patients with higher neuroticism and low conscientiousness were more likely to have a personality disorder. Therefore, it appears that the importance of these two personality traits is not specific to problematic internet use but is common in psychopathologies more generally.

The other three personality traits of the Big Five (ie, agreeableness, openness, and extraversion) did not correlate with problematic internet use in the sample in this study. This result might be explained by the fact that the recruited sample was very specific, including a higher proportion of users with more severe problems. Thus, it is tempting to hypothesize that emotional instability and low conscientiousness might be those personality factors that contribute to the maintenance of problematic internet use. However, prospective studies are needed to test this notion. In addition, it is worth noting that the previous correlational findings between problematic internet, openness, and extraversion were mixed, thus further studies are needed utilizing different samples.

Among the psychopathological symptoms, only obsessive-compulsive symptoms and depression were significant predictors of problematic internet use. These findings are in line with previous results [8,1,14,12,13,4,5,15]. In reviewing other addictive behaviors, there are some additional findings that reinforce the results of this study. For instance, in the case of compulsive buying, Maraz et al [60] found an increased level of obsessive-compulsive symptoms among addicted shoppers compared to nonaddicted shoppers. Moussas et al [61] investigated patients of a methadone maintenance treatment program, and depression and obsessive-compulsive symptoms were found to have the highest mean scores among all the symptoms. Similarly, in the case of methamphetamine users, obsessive-compulsive symptoms and depression were reported to have the highest levels among the psychopathological symptoms, especially for injectors (compared to methamphetamine users who used other routes of administration) [62]. Based on the previously mentioned findings, the association between problematic internet use and specific psychopathological symptoms is similar to the associations between other addictive behaviors and specific psychopathological symptoms (obsessive-compulsive symptoms and/or depression).

The correlational analyses showed that all the psychopathological symptoms correlated with problematic internet use (*r*=.268-.404). Additionally, using the Global Severity Index, the mediation model corresponded with the data. In this second model, the path coefficient of Global Severity Index to problematic internet use was higher compared to that of the individual symptoms in the first model. Overall, it appears that the level of psychological distress (as indicated by the Global Severity Index) is a more important factor regarding problematic internet use than the specificity of psychopathology.

Based on fit indexes, both models showed excellent fit to the data. Because the two models were not nested, they could not be compared directly. However, results of the two models appear to be convergent. More specifically, emotional stability only affected problematic internet use indirectly via psychopathological symptoms (regardless of the indexes used), whereas low conscientiousness only had a direct effect on problematic internet use.

The first mediation model examined in this study was partly in line with previous findings. According to Smits and Boeck [63], the behavioral inhibition system relates to neuroticism. In Park et al’s [37] mediation model, the behavioral inhibition system impacted internet addiction via depression, which reinforces the findings of the model here, low emotional stability had an indirect effect on problematic internet use (however, the direct effect was also significant). Regarding low conscientiousness, which negatively relates to the BASF (ie, the fun-seeking scale of the Behavioral Activation System) [63], Park et al’s study also found a direct association between BASF and internet addiction, similar to the findings of this study (between low conscientiousness and problematic internet use). However, in their model, the indirect effect was significant in the case of impulsiveness and anxiety, whereas this study did not show any significant indirect effects between conscientiousness, depression, and obsessive-compulsive symptoms. Based on the outcome of the second path analysis, it could be concluded that low emotional stability only affects problematic internet use indirectly (via psychological distress), whereas low conscientiousness affects problematic internet use directly.

Interpreting the models proposed here, two different types of problematic users might be considered in terms of personality. Problematic internet use has long been known as a heterogeneous phenomenon [64]. Chamberlain et al [65] found that problematic internet use exists with and without other impulsive/compulsive conditions. However, both impair quality of life. It might be assumed that there are different paths leading to problematic internet use depending on the user’s personality. One path could be when an individual with a high level of neuroticism tries to cope with their negative emotions by repeatedly using the internet more intensively (ie, compensatory internet use [66]). In such cases, the level of psychological distress (eg, depressive feelings) mediates between neuroticism and problematic internet use. Because neuroticism is associated (prospectively) to internalizing symptoms [67], a possible path from neuroticism into problematic internet could be via internalizing symptoms (ie, depression and anxiety).

The other path could be when an individual with a low level of conscientiousness becomes vulnerable to problematic internet use. Low conscientiousness is regarded as being disorganized, inefficient, careless, and sloppy because these characteristics equate to a deficit in the executive functions. This could also provide an explanation for the comorbidity with attention-deficit/hyperactivity disorder (ADHD) [68-70]. This theory is reinforced by previously reported findings. For example, Van Dijk et al [71] found that adults with ADHD showed a higher level of neuroticism and a lower level of conscientiousness than healthy controls. Additionally, Gomez and Corr [72] reported in their meta-analysis that inattentional symptoms were associated with low conscientiousness. Regarding internet gaming disorder (IGD), Argyriou et al [73] also conducted a meta-analysis and demonstrated that there was an association between IGD and impaired response inhibition. They conceptualized IGD as externalizing psychopathology. This is in line with Dong and Potenza’s [74] suggestion of a cognitive-behavioral model of IGD.

It should also be noted that obsessive-compulsive symptoms were assessed by items such as trouble remembering things, difficulty making decisions, and trouble concentrating. These items might also signal a deficit in the executive functions. However, this subscale was not a significant mediator variable between low conscientiousness and problematic internet use. In future research, it would be worth investigating impulsivity rather than obsessive-compulsive symptoms in the model, such as Park et al’s [37], or assessing executive functions with cognitive tests (eg, inhibitory control, decision making, shifting).

Nevertheless, in the model proposed here, the two paths were not independent from each other. This fact is consistent with other results and theories on different executive functions and the internalizing-externalizing dichotomy. Executive functions may also be divided into hot and cool components [75], in which hot executive functions are involved in highly motivating and emotional situations. Based on this differentiation, neuroticism is associated with the executive function [76,77]. Additionally, there is evidence that component facets of neuroticism and conscientiousness share a common neurological system, in which high neuroticism and low conscientiousness associate with lower scores on the executive function battery [78]. Similarly, internalizing and externalizing disorders are not independent from each other either [79]. Additionally, depression is associated not only with neuroticism but also with conscientiousness [80]. Hall et al [81] noted the role of both personality (primary conscientiousness and neuroticism) and executive functions in predicting health behavior patterns, which might underpin the relevance of the model presented here. However, the models only explained 32.5% and 28.9% of the variances of the PIUQ. Consequently, further research is needed to identify other important factors shaping the symptoms of problematic internet use. In addition to users’ individual personalities, situational, social, and environmental factors would also be worth investigating.

One of the major implications of the findings in this study is that clinicians should be educated about the possible cultural aspects regarding the associations of personality traits, psychopathological symptoms, and problematic internet use. Additionally, the findings of this study highlight the possibility of the differences between internet users concerning intensity of usage in the role of personal characteristics in developing problematic internet use.

Finally, it should be noted that this study has several limitations. First, the sample was nonrepresentative of internet users and included intensive internet users. More representative samples are needed in any replication. The sample was Chinese only and may not be representative of internet users in other countries. Therefore, future research should also include participants of other countries and cultures. The sample size was modest (although adequate for the statistical testing carried out) and future studies should try to recruit as large a sample as possible. It is also suggested that future studies should include samples with a more even distribution of females because the sample in this study was predominantly male. Gender differences can then be explored more thoroughly. Finally, the data were self-reported and open to well-known biases (eg, social desirability and poor memory recall). Taking these limitations together, generalization of the findings should be applied with caution. To gain reliable data, more objective reports should be added (eg, family members’ and friends’ reports on the internet user’s behaviors).

In conclusion, our study revealed the role personality plays in problematic internet use. However, to clarify the associations between different personality traits and internet addiction, further investigations are necessary that apply complex models including possible mediator variables such as psychopathological symptoms.

## Appendix

Multimedia Appendix 1 Correlations between the PIUQ and the subscales of BSI and BFI.

## Abbreviations

ADHD: attention-deficit/hyperactivity disorder
BSI: Brief Symptom Inventory
CFI: comparative fit index
IGD: internet gaming disorder
PIUQ: Problematic Internet Use Questionnaire
RMSEA: root mean square error approximation
TLI: Tucker-Lewis index
