# Supplementary material for: The Relationship Between Personality Traits, Psychopathological Symptoms, and Problematic Internet Use: A Complex Mediation Model
Source: J Med Internet Res. 2019 Apr 26;21(4):e11837. doi: 10.2196/11837 (PMC6658222; doi:10.2196/11837)
Supplement: Multimedia Appendix 1 [file jmir_v21i4e11837_app1.pdf]

Correlations between the PIUQ and the subscales of BSI and BFI.

Scales	2	3	4	5	6	7	8	9	10	11	12	13	14	15	16
1. PIUQ-9 Total	.268	.395	.312	.404	.308	.336	.274	.247	.315	.380	-.107	-.101	-.366	-.293	-.053
2. BSI Somatization	1	.628	.596	.682	.778	.623	.687	.654	.689	.833	-.257	-.245	-.253	-.289	-.145
3. BSI Obsessive-compulsive		1	.720	.738	.650	.700	.667	.638	.707	.841	-.343	-.157	-.402	-.353	-.143
4. BSI Interpersonal sensitivity			1	.762	.700	.679	.699	.705	.707	.844	-.271	-.125	-.277	-.375	-.056
5. BSI Depression				1	.763	.680	.700	.707	.802	.897	-.282	-.224	-.345	-.377	-.119
6. BSI Anxiety					1	.644	.758	.706	.776	.887	-.314	-.251	-.274	-.365	-.119
7. BSI Hostility						1	.708	.723	.683	.827	-.225	-.296	-.270	-.362	-.045
8. BSI Phobic anxiety							1	.728	.717	.855	-.355	-.269	-.265	-.395	-.137
9. BSI Paranoid ideation								1	.762	.846	-.301	-.285	-.241	-.439	-.033
10. BSI Psychoticism									1	.886	-.285	-.219	-.263	-.355	-.030
11. BSI Global Severity Index										1	-.333	-.260	-.344	-.428	-.105
12. BFI Extraversion											1	.465	.418	.474	.425
13. BFI Agreeableness												1	.454	.531	.449
14. BFI Conscientious-ness													1	.493	.475
15. BFI Emotional Stability														1	.220
16. BFI Intellect/Openness															1

Note. PIUQ-9 Total: Sum score of the Problematic Internet Use Questionnaire. BSI: Brief Symptom Inventory. BFI: Big Five Inventory.

Correlations above | .219 | are significant at P <.001.
